# Family relationships with pediatricians: the maternal views

**DOI:** 10.1016/j.rppede.2016.03.015

**Published:** 2016

**Authors:** Simone de Carvalho, José Martins

**Affiliations:** a Universidade Estadual de Campinas (Unicamp), Campinas, SP, Brazil

**Keywords:** Pediatrician, Child guidance, Women's group

## Abstract

**Objective:**

To analyze the perception of pediatric guidelines by mothers at the time of consultation in private offices, in order to know how they assimilate, process and use the information received from the pediatricians.

**Methods:**

Data collection was carried out by a questionnaire sent to participants by a total of 200 mothers from a virtual community in social networks participated in the research. The answers were transcribed using the Discourse of the Collective Subject method. The analyses were supported by the research qualitative perspective, from the viewpoint of the social representation theory.

**Results:**

Three categories were obtained through data analysis: (1) assessing the pediatric guidelines, (2) confronting theory and practice and (3) developing a critical view of the pediatric guidelines. These categories have elucidated that the level of knowledge of pediatric issues by mothers and their ability to use them when making decisions about the care of their babies, have a direct association between following or not the pediatric guidelines.

**Conclusions:**

The mother's decision on following the pediatrician's recommendations depends on two main factors: (a) certification of the updated and proven recommendations, according to the official health agencies; (b) support and recognition by the pediatrician of the maternal empowerment during the follow-up process. The mothers' practice of accessing knowledge through social networks hinders the pediatric monitoring.

## Resumo

**Objetivo:**

Analisar a percepção das orientações pediátricas pelas mães na ocasião do atendimento em consultórios particulares, para conhecer de que maneira assimilam, processam e usam as informações recebidas de seu pediatra.

**Métodos:**

A coleta de dados foi feita por questionário enviado às participantes. Participaram da pesquisa 200 mães de uma comunidade virtual nas redes sociais. As respostas foram transcritas por meio do método do discurso do sujeito coletivo. As análises foram respaldadas na perspectiva qualitativa de pesquisa, sob a ótica da teoria das representações sociais.

**Resultados:**

Obtiveram-se três categorias por meio da análise de dados: (1) avaliação das orientações pediátricas, (2) confronto da teoria e prática e (3) desenvolvimento de um olhar crítico acerca das orientações pediátricas. Tais categorias elucidaram que o nível de conhecimento de temas pediátricos por parte das mães e a sua capacidade de usá-los na tomada de decisões sobre os cuidados dos seus bebês apresentam uma relação direta entre seguir ou não as orientações pediátricas.

**Conclusões:**

A decisão das mães quanto a seguir as recomendações do pediatra depende de dois fatores principais: (a) certificação das recomendações atualizadas e comprovadas, de acordo com os órgãos oficiais de saúde; (b) apoio e reconhecimento por parte do pediatra do papel materno durante o processo de acompanhamento. A prática do acesso ao conhecimento das mães por meio de redes sociais dificulta o acompanhamento pediátrico.

## Introduction

In our society, the pregnant woman is seen as a sick individual,^1^ requiring frequent medical care from conception to delivery. The Western method of Medicine is still associated with detachment, impersonality, objectivity and the care provider's authority. This perspective directly influences the way this woman sees herself and in many cases affects her self-confidence and capacity to manage the maternal processes, including delivery, breastfeeding and care provided to the baby.^2^

The literature has been increasingly discussing the empowerment processes^3^ and their applicability to motherhood. Maternal empowerment can be understood as women's achievement toward strengthening their personal autonomy, which may occur individually or collectively, making them capable of self-managing their maternal dilemmas.^4^

Empowerment in the health area is seen by its professionals as a support tool in health self-control processes by patients. It has been shown that women who manage or are directly responsible for the care of their babies reach, as a result, a significant improvement in child health quality.^5^

The attainment of knowledge on baby care by the mother, based on her own maternal experiences and acquired knowledge, has a direct effect on her decision to follow or not the advice she receives from this professional.^6^ This decision is based on internalized suppositions from experiences and knowledge acquired by this mother during her own existence and experiences shared with other women, especially when she feels insecure about the recommendations she received from the pediatrician. In this sense, pediatricians who show appreciation for these maternal experiences and thus reaffirm the mother's personal beliefs will be more successful in their professional performance.

This study considers the maternal empowerment as any behavior that gives mothers a positive and informed control regarding their decisions on the care of their babies' health, which result in what might be called good pediatric practices. The objectives of the study include: to understand the meanings of discourses through maternal practices, to associate the meaning assigned by them to such behavior and their perceptions of pediatric care in this interaction.

Therefore, given the scarcity of data on the impact of maternal empowerment in promoting and improving maternal and child health, we decided to assess the perceptions of mothers about pediatric care, studying in depth the factors that motivate this practice among mothers, as well as the support available on the internet.

## Method

This is a descriptive exploratory study, which allowed us to interpret the discourses of the mothers participating in a virtual motherhood support group, regarding their perceptions of pediatric care and the consequent decisions regarding the health of their babies, characterizing the type of behavior that is defined as maternal empowerment (ME). The analyzed virtual community on Facebook has been active for five years as a support group that guide and share experiences daily with thousands of mothers through the Internet, relying on the voluntary participation of approximately 50,000 mothers living in several Brazilian cities. The community's goal is maternal empowerment in the virtual network.

The main reason that leads mothers to seek the community, in general, is experiencing some difficulty in the postpartum period, especially regarding breastfeeding. The community usually follows this mother in the daily dynamics of involvement and care of her baby and she participates in the support groups according to her needs, remains a member of these groups - monitors the daily publications and actively participates in community life - through debates, supporting other mothers and sharing personal experiences related to their maternal processes. The fruitfulness and variety of this virtual support network allows us to understand the dynamics of this relationship and its practical consequences in child care and thus, meet the goals of this research.

This study was approved by the Institutional Review Board of Faculdade de Ciências Médicas da Unicamp, under protocol number 793,995. All participants signed the Informed Consent form after reading and agreeing to the objective of the study. The anonymity of participants was guaranteed. The mothers were selected from the community through a poll generated by the social network system and those interested in participating in the study provided their email address through a private message, individually.

Data were collected from September 19 to October 9, 2014 through a semi-structured questionnaire sent to participants by e-mail, aiming to assess the mothers' perceptions about the pediatric guidelines at the time of consultation and on the consequent judgment of value they developed from this interaction with the physician. The first part of the questionnaire contained questions that characterized the study population. In the second part, two categories were created to investigate the history of pediatric care of mothers in the postpartum period, with six sub-categories for the topic analysis. In the last sub-category, the mothers had space to justify their answers, which assisted in the analysis of data obtained through the 200 completed questionnaires.

Mothers were approached by formal invitation through a poll previously created by the tool on the Facebook social network. The second phase consisted in the collection of questionnaires completed by the mothers. Of the 350 emails sent, exactly 200 emails were returned, appropriately filled out and signed. Of the remaining 150 mothers, 26 refused to participate and 124 did not answer e-mail and the discourse of the collective subject was applied to the 200 responses received from the questionnaires.

The qualitative data were classified according to the comprehensive study of the discourses, which indicates a reality of experiences lived by these mothers and their accuracy leaves no doubt regarding the reliability of reproduction of variables, for which readers are able to recognize.^7^

## Results

We sought to describe, through the mothers' responses to the questionnaire, their perceptions of the pediatric guidelines at the time of the consultations and the consequent judgment of value they created based on this interaction with the health care professional. This study resulted in three categories: (1) evaluation of pediatric guidelines, (2) confrontation of theory and practice and (3) developing a critical view of the pediatric recommendations.

### Evaluation of pediatric guidelines

"I followed the pediatrician's recommendations because they were clear and according to my convictions and compatible with the information I researched in the mothers' groups for guidance on social networks. I felt very insecure in the first moments and I trusted her because of her professional experience and correct recommendations, which I considered consistent. The recommendations were clear, precise and it was very important to receive guidance that went against what our family preached, and so they also learned from the guidelines and have come to respect the recommendations we received, in addition to the assistance provided by the virtual groups. Nowadays she knows me well and knows what my priorities are." **(DSC 1)**

The evaluation of pediatric guidelines for the mothers of this study is based on the figure of the pediatrician in relation to their professional experience and credibility as an expert in the area, but also the compatibility of their recommendations with their personal knowledge and their maternal perceptions. The pediatrician's action in recognizing their priorities and sharing the maternal impressions supports the mother's decision-making, both in her choice and the decision to continue the follow-up. The dynamics of everyday knowledge shared through the mothers' socialization, based on readings and studies available on the Internet and virtual discussion groups, influences their maternal management processes. It is based on this collected and internally systematized information that mothers filter the information, which allows them to decide whether or not to follow the received instructions.

### Confrontation of theory and practice

"I did not follow all the pediatrician's recommendations because I had read much about the subject; I always followed my natural mother's instinct and I chose what I thought was best. What I considered unnecessary according to my research and personal experience I did not follow, as it did not match what I had learned from the groups I participate in the internet. In fact, I would not say all, but most, and at times I resorted to the opinion of other mothers. I used the pediatrician's recommendations as an addendum to my decisions, because I knew what I needed to do, that is, I knew I could have other options, and not simply accept them without further investigation. I followed the recommendations I considered consistent with the current practice of Pediatrics and the WHO recommendation." **(DSC 1)**

The constant search of mothers for additional sources of knowledge in virtual groups of their interest can be understood as a conscious effort to compare the opinion of the health professional, balancing it with the acquired knowledge and their own perceptions of motherhood. In this sense, the pediatric recommendations become just one of many sources of support for making decisions about the care of their babies.

"It is the fact of thinking a little different than the professional. I think it is very complex to receive general recommendations from the professional and some of them go against what I saw as correct and others that did not make sense to me. They are treating the standard child, not my child, and that made me seek more information on childhood-related issues and seek other professionals. After the group, I became more critical and started questioning. Being very honest, it is difficult for me to fully trust the recommendations by pediatricians because they do not always understand my choices… It is worth mentioning that I went to five pediatricians until I found one that came closer to my expectations, because they did not fit with my view on maternity; 99% of the pediatricians I visited prescribed complementary feeding and industrialized foods." **(DSC 1)**

Appreciating the importance of these mothers' maternal empowerment practice becomes vital to understand the decision-making process regarding the care of their babies, with their interaction with peers and health professionals that treat their babies. From the perspective of the interviewed mothers, being empowered means being in control of their maternal processes based on this learned behavior.

"Although the pediatrician provides all the information, we sometimes follow our intuition and do what works at home. Because I am the mother and I know what my baby needs, I've always researched a lot about my doubts and often did not have the correct answer from the said pediatrician. I used my common sense and sensibility; in theory I think it is easy and simple, but in practice it is quite different and in some moments I preferred to follow my intuition. Most of the time, I ask other friends who are mothers, other pediatricians and the internet before I follow certain recommendations. I try to consider everything, what I know and the pediatrician's clinical evaluation. I very often do what my heart tells me to." **(DSC 1)**

The multiple impressions about the received pediatric guidelines, which are shared in the virtual support group, demonstrate a confrontation between theory and practice; confirm the maternal observation and intuition as ideal to convey to the pediatrician the specific needs of their baby and the appropriateness of care desired by these mothers.

### Development of a critical view of the pediatric recommendations

"I do not agree with certain procedures and recommendations; I investigated and saw that the pediatrician was out of date. They were inadequate and outdated when compared to the recommendations of national and international societies. I had great confrontations with the pediatrician to be able to maintain exclusive breastfeeding for six months and did not introduce other foods early (at four months) as recommended. After this consultation, I switched doctors. First because of the absurd recommendations, second because of the lack of breastfeeding support and third because I stopped believing in everything he said, because they were always too vague. I looked for information on the internet and once again disregarded the pediatrician's recommendation. I realize that the ideal is a pediatrician in whom we trust to eliminate all our doubts, but we are living in a time when the Brazilian medical profession is going from bad to worse, more concerned with the number of consultation than their quality." **(DSC 1)**

**Figure 1:**
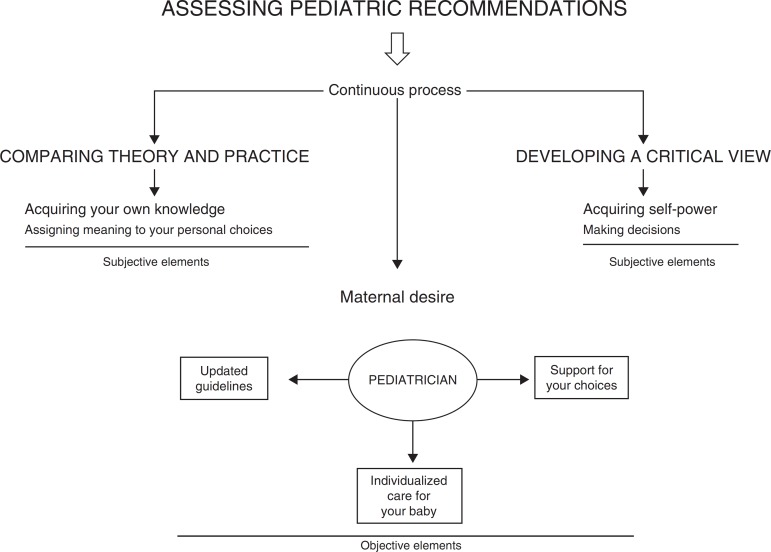
The meaning of the pediatric recommendations at the time of the consultation according to the concept of maternal empowerment.

The mother's decision to follow the pediatrician's recommendations is supported both by what she hears from the pediatrician and by her knowledge, based on her maternal experiences. Since the mother is in a constant exercise to confront what the pediatrician says and what is *her own* truth, the appropriation of her acquired self-power is disclosed and the action of decision-making based on the evaluations of these two distinct realities result in the practical behavior of this relationship, especially when there is lack of support and recognition by the pediatrician. These categories were understood as particularities of the discourse of these mothers that belonged to the assessed maternal group. The results of the meaning of pediatric recommendations at the time of the consultation for the mothers participating in this study were described and exemplified through a flowchart (Fig. 1).

## Discussion

The cyberspace has become a place of interaction, where mothers can openly express their emotions about motherhood. For Porter,^8^ mothers are increasingly turning to the Internet seeking these network-shared actions.^9^^,^ 10 The term "empowerment"^11^ is relatively new and has been studied in relation to its applicability in people's self-management acquisition. Social representations^12^ related to the mothers' interactivity and discursive lines demonstrate the practice of a contagious empowerment repertoire among the mothers, through their own interpretations and perceptions of reality experienced in pediatric offices, in a constant action of developing views and perceptions to reconstruct and modify them. Virtual groups of mutual support, awareness and sharing experiences and perceptions provide the basic elements that allow the maternal empowerment described herein.

Especially in the health area, the knowledge of maternal empowerment becomes important for professionals to allocate time for the mother to make her own decisions regarding her baby's care and to assess which information is relevant, in order to verify the practical applicability and agreement with the provided recommendations, - the bioethics of care - which may ultimately influence the mother's decision to follow or not these recommendations.^13^^,^ 14

In the results of the study by Bonvicini,^15^ which recorded 1800 interactions of discourses of 170 doctors with their patients, it was observed that medical empathy is essential for the patient to comfortably express important concerns and problems. Such studies have shown that physicians recognize the presence of a communication gap, specifically in the management of behavioral and emotional reactions and the need for the involvement of a comprehensive educational program in relation to counseling, which could result in a positive change in their empathy expression. On the other hand, pediatricians are burdened by a service based on productivity and that does not leave them enough time to talk to the mother, an essential part of the pediatric consultation.

During the maternity leave period, usually the first four months of the baby's life, mothers actively participate in virtual groups seeking help, guidance and mainly immediate support due to the availability of time and easy access to virtual social networks. Mothers also constantly share in groups what many pediatricians recommended regarding the same subject; bring their questions, add acquired knowledge on that issue through virtual search, evaluate such information based on their personal experiences and usually in the following consultation, discuss with their pediatrician what they have discovered or choose to change pediatricians and even discontinue the care, if their perception is negative in relation to the acquired knowledge. The mothers show they are able to assess whether such information is safe and show increasingly more criticism regarding the available content and choose reliable sites; this was made possible through the emergence of virtual social networks. According to Bartlett,^16^ the mother's trust of the pediatric care is strengthened when her personal experiences and her personal empowerment are confirmed and recognized. Maternal empowerment on the internet is a powerful and facilitating tool of maternal and child health.

According to the data, there is a recurring behavior in most of the mothers' discourses about the constant consideration regarding the pediatric recommendations received by them and knowledge of the recommendations issued by national and international health agencies, through free access to this information on the Internet. In the discourse of the mothers participating in the study, the finding of the majority of negative responses - that the pediatric recommendations given to the mothers were outdated and not consistent with their acquired knowledge - hindered the relationship of care and follow-up of their babies. This was due to the fact that there was distrust between the information that solved their questions and the confrontation of the non-confirmation of these questions at the time of the pediatric consultation, a hypothesis raised in the study by Berkel.^17^

By offering support to the mother and listening to her, on the part of the health professionals, it can be concluded that maternal empowerment is an important tool for the mothers to exercise their motherhood while aware of their daily experience in the observation and care of their babies. No one better than the mother knows her baby as well as she does - what is happening with the child and its peculiarities. The mother is, therefore, an assiduous and crucial observer as a support figure for the quality of care provided by the pediatrician. In this study, we identified the process of maternal empowerment as supportive of this relationship, in which the first step is the mothers' evaluation of received recommendations; the second, the confrontation between theory and practice based on their personal empowerment and, finally, the development of a critical view through the acquisition of knowledge, now acquired through the virtual support groups.

The limitations of this study are related to the need for the creation of a specific tool to validate the degree of maternal empowerment and the carrying out of future studies to reinforce the results of the present one. In the analysis of women from eight countries, empowerment indicators are not all similar in terms of methodology and thus, the creation of a quantification of empowerment indicators for a correct assessment is necessary, as the incapacity of a purely statistical approach would have conceptual implications, according to Kabeer.^18^

Knowing the maternal empowerment behavior and its practice is relevant to understand why mothers are increasingly using social networks to share their perceptions, experiences and their knowledge about maternity and pediatric care. It is worth mentioning that children's health is closely related to the mothers' decisions according to the perceived pediatric instructions.
